# Extracellular NM23 Protein as a Therapeutic Target for Hematologic Malignancies

**DOI:** 10.1155/2012/879368

**Published:** 2011-09-19

**Authors:** Junko Okabe-Kado, Takashi Kasukabe, Yasuhiko Kaneko

**Affiliations:** Research Institute for Clinical Oncology, Saitama Cancer Center, Komuro 818, Ina-machi, Saitama 362-0806, Japan

## Abstract

An elevated serum level of NM23-H1 protein is a poor prognostic factor in patients with various hematologic malignancies. The extracellular NM23-H1 protein promotes the *in vitro* growth and survival of acute myelogenous leukemia (AML) cells and inversely inhibits the *in vitro* survival of normal peripheral blood monocytes in primary culture at concentrations equivalent to the levels found in the serum of AML patients. The growth and survival promoting activity to AML cells is associated with cytokine production and activation of mitogen-activated protein kinases (MAPKs) and signal transducers and activators of transcription (STAT) signaling pathways. Inhibitors specific for MAPK signaling pathways inhibit the growth/survival-promoting activity of NM23-H1. These findings indicate a novel biological action of extracellular NM23-H1 and its association with poor prognosis. These results suggest an important role of extracellular NM23-H1 in the malignant progression of leukemia and a potential therapeutic target for these malignancies.

## 1. NM23 Expression and Hematologic Malignancies

The NM23 gene was identified by differential hybridization of a cDNA library with total RNA extracted from slightly and highly metastatic melanoma cell lines [1]. The NM23 gene has been identified as a family of genes encoding different isoforms of nucleoside diphosphate kinase (NDPK) [2]. NM23 genes play critical roles in cellular proliferation, differentiation, oncogenesis, and tumor metastasis, and the mechanisms of these pleiotropic effects are not well understood [3, 4]. Ten isotypes of the human NM23 gene have been identified [5]. Among these, only NM23-H1 and NM23-H2 have been studied extensively in human cancers. The level of NM23-H1 expression is inversely correlated with the tumor's metastatic potential in experimental rodent cells and in human tumors, such as breast, ovarian, cervical, and gastric cancer, hepatocellular carcinoma, and melanomas [4]. Exogenous overexpression of NM23-H1 reduces the metastatic potential of multiple types of cancer cells and suppresses *in vitro* tumor cell motility and invasion [6]; therefore, NM23-H1 is implicated in the regulation of metastasis in a variety of human cancers, and its overexpression predicts a favorable patient prognosis. In contrast, elevated NM23-H1 expression is related to a more aggressive disease in neuroblastoma and many hematologic malignancies [7–11]. The significance of NM23-H1 overexpression as a prognostic factor is dependent on tumor cell types although the mechanism of this discrepancy is unknown.

**Figure 1:**
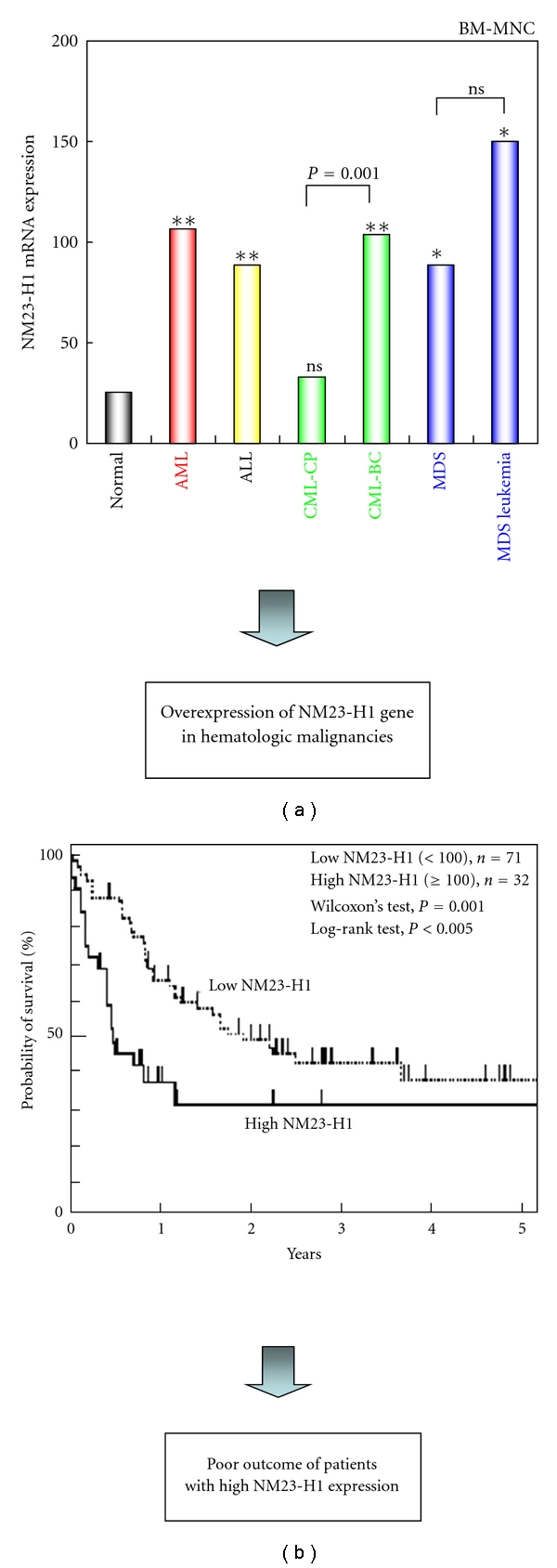
Clinical significance of NM23-H1 expression in hematologic malignancies. (a) Overexpression of NM23-H1 gene in hematologic malignancies [10, 20, 31]. Quantitative RT-PCR analysis on NM23-H1 mRNA in human bone marrow mononuclear cells (BM-MNC) in normal (*n* = 5), AML (*n* = 110), acute lymphoblastic leukemia (ALL, *n* = 9), and chronic myelogenous leukemia in the chronic phase (CML-CP, *n* = 9), CML in blast crisis (CML-BC, *n* = 7), myelodysplastic syndrome (MDS, *n* = 9), MDS overt leukemia (*n* = 5). The mRNA levels were normalized for *gapdh* mRNA. The positive control (the index = 100) is represented by RNA extracted from the human leukemia cell line (HEL). Mann-Whitney's *U* test (versus normal). *P < 0.05, **P < 0.001. (b) Overall survival curves of patients with AML, according to NM23-H1 expression level [31]. High NM23-H1 (>100 index) patients (*n* = 32, solid line) had a worse prognosis than low NM23-H1 (≤100) patients (*n* = 71, broken line).

We previously reported that a nondifferentiating myeloid leukemia cell line produced differentiation-inhibiting factors [12, 13]. We purified one of these factors as a homologue of mouse NM23-M2 [14]. The NM23-H1 gene was overexpressed in various hematologic neoplasms, including AML, acute lymphoblastic leukemia (ALL), chronic myelogenous leukemia in blastic crisis (CML-BC), and myelodysplastic syndrome (MDS), more than in normal blood cells (Figure 1(a)) [10]. The progression of CML was accompanied by the overexpression of NM23-H1 mRNA [15]. This increase in NM23-H1 was observed not only in leukemia, but also in malignant lymphoma. It has been reported that high-grade non-Hodgkin's lymphoma and Hodgkin's lymphoma exhibited significantly higher levels of NM23-H1 expression than low-grade non-Hodgkin's lymphoma [16–19]. NM23-H1 gene was overexpressed in AML cells, and a higher level of NM23-H1 expression was correlated with a poor prognosis in AML (Figure 1(b)) [10, 20, 21]. Multivariate analysis of putative prognostic factors revealed that elevated NM23-H1 mRNA levels significantly contributed to the prognosis of patients with AML [10]. NM23-H1 and NM23-H2 are highly expressed in normal CD34^+^ hematopoietic progenitors but are downregulated during normal hemopoietic maturation [22]. These genes are also downregulated during *in vitro* differentiation of AML line cells [23, 24]. These findings suggest an important role for NM23-H1 and NM23-H2 in controlling hemopoietic differentiation and leukemic progression [22–24]. Taken together, these results indicate that the expression of NM23 is downregulated during hematopoietic maturation, and its overexpression is found in many hematologic malignancies and predicts poor treatment outcome of patients with AML (Figure 1).

## 2. Clinical Significance of Extracellular NM23-H1 Protein in Hematologic Malignancies

NM23 has no secretion signal peptide but is nonetheless detected in conditioned medium of some tumor cell lines and in body fluids [14, 25–28]. The mechanisms by which NM23-H1 protein is secreted into the extracellular environment are unclear. Recently, Keller et al. [29] showed a new pathway for the secretion of many inflammatory response proteins without signal sequences. This unconventional secretion required the catalytic activity of caspase-1 and could rapidly release a wide variety of proteins involved in trigger detoxification, tissue repair, and cell survival. Furthermore, an exosome-associated export pathway of a number of proteins without signal sequences from the cells is reported [30]. It would be interesting to examine whether these new secretion pathways secrete NM23 protein; however, unlike secretion, it might be the release of NM23 protein by dying tumor cells overexpressing NM23.

**Figure 2:**
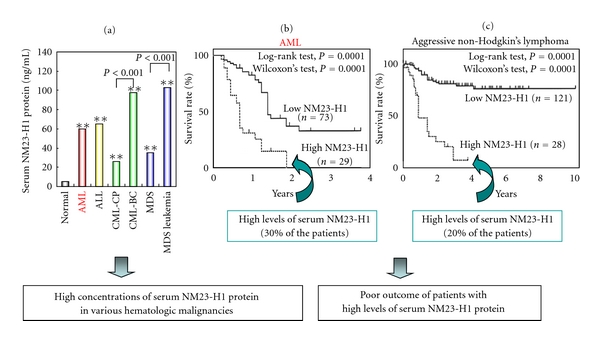
Clinical significance of extracellular NM23-H1 protein in hematologic malignancies. (a) Serum NM23-H1 protein levels in normal healthy control and hematologic malignancies [31]. Normal control (*n* = 21), AML (*n* = 102), ALL (*n* = 6), CML-CP (*n* = 13), CML-BC (*n* = 9), and MDS (*n* = 12), MDS overt leukemia (*n* = 6). Mann-Whitney's *U* test versus control, **P < 0.001. (b) Overall survival curves of patients with AML [32]. High-NM23-H1 (≥80 ng/mL) patients (*n* = 29) had a worse prognosis than low-NM23-H1(<80 ng/mL) patients (*n* = 73). (c) Overall survival curves of patients with intermediate and high-grade non-Hodgkin's lymphoma [11, 25]. High-NM23-H1 (>80 ng/mL) patients (*n* = 28) had a worse prognosis than low-NM23-H1(<80 ng/mL) patients (*n* = 121).

We determined the serum level of NM23-H1 protein by enzyme-linked immunosorbent assay (ELISA) and assessed the association between this level and the clinical outcome of patients with hematologic malignancies [17, 25, 31, 32]. As shown in Figure 2(a), serum NM23-H1 levels were significantly higher in all of these hematologic malignancies used than in a normal control [31, 32]. The 102 patients with AML were divided into two groups with different serum NM23-H1 levels, to compare the overall survival of the two groups. All the patients with levels higher than 80 ng/mL died within 2 years. The 2-year survival rates for the high-NM23-H1 (≥80, *n* = 29) and low-NM23-H1 (<80, *n* = 73) groups were 0% and 33.3%, respectively (Figure 2(b)). The univariate analysis showed that unfavorable prognostic factors for overall survival were WBC count over 50,000/*μ*L, LDH level over 5× normal, and NM23-H1 protein level over 80 ng/mL. Multivariate analysis using Cox's proportional hazard model showed that serum NM23-H1 level (≥80 ng/mL) was the strongest unfavorable factor, followed by WBC count and LDH. Thus, the elevated serum NM23-H1 levels significantly contributed to the prognosis of AML patients [32]. The 149 patients with aggressive (intermediate- and high-grade) non-Hodgkin's lymphoma were divided into groups with different NM23-H1 levels at a cutoff value of 80 ng/mL. The 3-year survival rates for the high (≥80, *n* = 28) and low NM23-H1 groups (<80, *n* = 121) were 6.7 and 76.4%, respectively (Figure 2(c)). These results suggest that an elevated serum NM23-H1 concentration predicts a poor outcome of aggressive non-Hodgkin's lymphoma [25]. The prognostic ability of serum NM23-H1 protein was confirmed by examining a number of patients with various types of malignant lymphoma in our study involving a number of different institutions and numerous case studies [11, 17–19, 32]. 

 Extracellular NM23 proteins have been reported in the conditioned medium of some tumor cell lines, in body fluids, and on the cell surface in tumor cell lines [14, 22, 26–28, 35, 36]. High concentrations of NM23 protein are found in the serum and body fluid of patients with tumors overexpressing NM23, and it is strongly suggested that serum NM23 protein is derived from tumor cells [18, 31, 32]. Once again serum NM23-H1 levels were significantly higher in patients with all hematologic malignancies tested than in normal/healthy controls (Figure 2(a)), and an elevated serum NM23-H1 protein concentration predicted a poor outcome of AML (Figure 2(b)) and various types of malignant lymphoma (Figure 2(c)) [11, 17–19, 32]. These results suggest that extracellular levels of NM23-H1 play an important role in clinical outcome in patients with AML and malignant lymphomas.

## 3. Biological Functions of Extracellular NM23-H1 Protein

The mechanisms by which NM23-H1 protein is secreted into the extracellular environment and affects the outcome of patients are unclear. Very little information is available concerning extracellular expression and function although many studies have examined the expression of intracellular NM23 proteins; therefore, we focused on extracellular NM23-H1 protein derived from tumor cells, because its clinical significance is higher than that of intracellular overexpression [31], and the elevated extracellular expression of NM23-H1 has not been found in normal healthy plasma [25]. To demonstrate the clinical importance of extracellular NM23-H1 protein as a therapeutic target of patients with hematologic malignancies, we surveyed the biological functions of extracellular NM23-H1 protein. First, we investigated the extracellular functions of recombinant NM23 (rNH23) proteins on the survival and growth of normal and leukemic PBMNC and their association with the poor prognosis of AML patients. 

### 3.1. Effect of Extracellular NM23 Protein on the *In Vitro* Growth/Survival of Primary Cultured AML Cells

**Figure 3:**
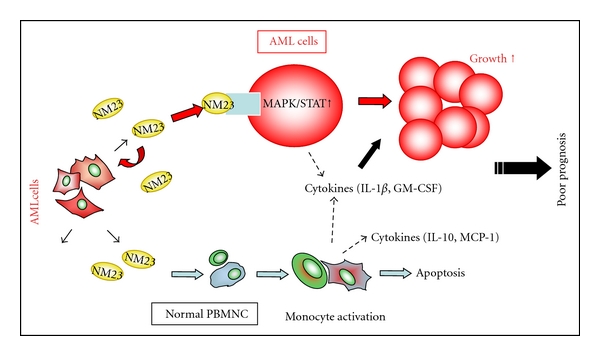
Extracellular function of NM23-H1 protein derived from tumor (AML) cells on primary cultured normal PBMNC and AML cells. Extracellular NM23-H1 protein promotes the growth/survival of primary AML cells mediated by MAPK activation, STAT activation, and cytokine release [33]. On the other hand, extracellular NM23-H1 protein affects normal PBMNC survival, activates monocytes, and induces cytokine production [34]. Some of these cytokines, especially GM-CSF and IL-1*β*, directly promote the survival/growth of primary cultured AML cells. Moreover, NM23-H1 induces immunosuppressive cytokine, such as IL-10. Therefore, the cytokine-inducing effect of extracellular NM23-H1 protein on normal PBMNC may be associated with a poor prognosis in AML. Taken together, these observations suggest that extracellular NM23-H1 may play an important role in the malignant progression of leukemia and that inhibitors of extracellular NM23-H1 protein or inhibitors of extracellular functions of this protein should be evaluated for their treatment.

To investigate the potential pathological link between the elevated serum level of this protein and poor prognosis, we examined the extracellular functions of rNM23 protein on the *in vitro* growth/survival of primary cultured AML cells. rNM23-H1 protein promoted the *in vitro* growth/survival of AML cells at concentrations equivalent to the levels found in AML patients. This finding indicates a novel extracellular function of NM23-H1 and its potential link with poor prognosis (Figure 3). Both rNM23-H1 and rNM23-H2 promoted the growth/survival of AML cells; therefore, this activity of rNM23 is independent of H1/H2 isotypes. The mutant NM23-H1^His^ protein, which does not have any NDPK activity, also promoted the growth/survival of AML cells. These results indicate that the activity of NM23 is independent of its NDPK activity [33]. How extracellular NM23-H1 protein promotes the growth/survival of AML cells remains unclear. We examined cytokine levels in the conditioned medium (CM) of AML cells treated with or without rNM23-H1, using cytokine antibody array and ELISA. Various cytokines and chemokines were detected in 48 h CM of AML cells growth/survival promoted by rNM23-H1 [33]. These cytokines included TNF*α*, IL-1*β*, IL-6, IL-8, I-309, IL-10, GM-CSF, and RANTES. The cytokine-inducing activity of rNM23-H1 was associated with its growth/survival-promoting activity for AML cells. Although the patterns of cytokine induction are different among cases, cytokines known as a growth factor for AML cells, such as GM-CSF and IL-1*β*, were induced in CM of NM23-sensitive cases but not NM23-unresponsive cases. Moreover, the induced-cytokine concentrations reached physiologically effective levels. To investigate the correlation between cytokine-inducing activity and the growth/survival-promoting activity of rNM23-H1, we tried to inhibit the cytokines using specific antibodies (anti-TNF*α*, anti-IL-1*β*, and anti-IL-6 antibodies). All these antibodies alone or some combinations tested partially inhibited the rNM23-H1-induced growth/survival of AML cells. Anti-GM-CSF antibody also inhibited both GM-CSF-induced and rNM23-induced growth/survival of AML cells. These results suggest that the growth/survival-promoting activity of this protein may be partly mediated by the induction of these cytokines (Figure 3). We next investigated the NM23-induced-signal transduction pathways relating with cell proliferation, survival, and cytokine production, namely, MAPK (p38, Erk1/2, JNK), STAT, AKT/PI3K, and NF*κ*B in AML cells. Of these pathways, extracellular rNM23-H1 activated MAPK (p38, Erk1/2) and STAT, but not others [33]. STATs are known as key proteins playing roles as signal messenger and transcription factors that participate in normal cellular responses to cytokines. The constitutive activation of STAT3 has been reported to be associated with a wide variety of human malignancies containing AML. rNM23-H1 increased the total amount of STAT1 protein and the phosphorylation of Tyr and Ser. STAT3 was phosphorylated on Tyr705 in the absence of rNM23-H1, but Ser727 of STAT3 was also phosphorylated in the presence of rNM23-H1. Ser phosphorylation of STAT3 has been reported to be required for maximal transcriptional activity [37]. STAT5 (Tyr694) was also activated by rNM23-H1 [33]. The individual signaling pathways induced by rNM23-H1 protein were blocked in AML cells using specific pharmacological inhibitors, SB202190 and SKF86002 for p38 MAPK, PD98059 for extracellular signaling kinase (ERK, also known as MEK), and Curcumin for STAT3 [38]. These inhibitors suppressed the rNM23-induced growth/survival promotion of AML cells [33]. These findings indicate that the activity of rNM23-H1 is associated with the activation of these signaling pathways in AML cells [33]. MAPK/STAT activation by conventional growth factors takes only a few minutes; however, the activation of these signaling pathways by rNM23-H1 required a longer time than various mitogens [33, 39]; therefore, it might be an indirect activation rather than a direct activation by NM23 molecules. Taken together, these observations suggest that extracellular NM23-H1 may play an important role in the malignant progression of leukemia, and the inhibitors of extracellular NM23-H1 protein or inhibitors for the signaling pathways activated by extracellular NM23-H1 should be evaluated for their potential treatment as an approach to these malignancies (Figure 3). 

 Lilly and his colleagues reported heterogeneity in the ability of AML samples to bind and respond to extracellular NM23-H1 [40]. The authors offered evidence that binding was essential for support survival. Although rNM23-H1 promoted the survival of the most primitive blasts within responding AMLs, it was not these cells that actually bound the protein. Instead, rNM23-H1 bound to more mature CD34^low^/CD34^−^ and CD11b^+^ cells, showing an indirect survival benefit of rNM23-H1 on primitive blasts. Collectively, these results show that NM23-H1 preferentially binds to the more mature cells of the AML clone that are CD34^low^/CD11b^+^. However, the survival of both the more mature (CD34^low^/wCD11b^+^) and immature (CD34^+^/CD11b^−^) cells is enhanced. In support of this finding, the survival of purified blast cells was enhanced by conditioned medium (CM) of more mature cells from the clone that had been stimulated by rNm23-H1. Levels of IL-1*β* and IL-6 in rNM23-H1 CM mirrored the potency of the CM to promote blast cell survival. These data indicate that NM23-H1 indirect survival affects the CD34^+^/CD11b^−^ cells, by inducing supportive cytokine release from the more mature CD34^low^/CD11b^+^ cells. The authors offered the first evidence of novel crosstalk between cell populations within the tumor (AML) clone. Moreover, these findings have implications for the role of NM23-H1 in AML and its use as a prognostic marker, well coinciding with our results (Figure 3).

### 3.2. Effect of Extracellular NM23 Protein on the *In Vitro* Growth/Survival of Primary Cultured Normal Peripheral Blood Mononuclear Cells (PBMNCs)

We examined the extracellular effects of rNM23-H1 protein on the *in vitro* survival of primary cultured normal PBMNC. rNM23-H1 inhibited the survival of PBMNC at concentrations equivalent to the levels found in the serum of AML patients [34]. The rNM23 responsible adherent cells were CD68-positive and nonspecific-esterase- (NSE-) positive monocytes. On the other hand, rNM23 did not inhibit, rather slightly stimulated, the survival of nonadherent PBMNC (mainly CD3-positive lymphocytes). These results indicate that extracellular rNM23-H1 protein affects the *in vitro* survival of normal immune cells, such as monocytes. The inhibitory effect of this protein on normal monocytes may be associated with the poor prognosis of hematologic malignancies, since monocytes/macrophages also play a crucial role in the immune system.

 Moreover, rNM23-H1 protein promoted the production of various cytokines and chemokines, including proinflammatory cytokines in normal PBMNC, especially in monocytes [34]. By using a human cytokine array system, we analyzed NM23-regulated cytokines in CM of PBMNC treated with rNM23-H1. Cytokine arrays showed that the expressions of inflammatory cytokines (IL-1*β*, IL-6, IL-8, GM-CSF, and MCP-1) were significantly induced in CM of rNM23-H1-treated cells. It has been known that inflammation is an important component of the tumor microenvironment although the mechanisms through which immune cells might promote tumorigenesis are unclear [41]. Some cytokines induced by rNM23-H1 such as GM-CSF and IL-1*β*, practically and directly promoted the growth/survival of primary cultured AML cells (Figure 3). The cytokine arrays also showed that rNM23-H1 enhances the production of MCP-1 and IL-10 by normal PBMNC [34]. It has been described that MCP-1 increases recruitment of tumor-associated macrophages (TAMs), leading to a higher degree of angiogenesis [42]. TAMs generally express an M2-like phenotype [43], which is characterized by high IL-10 expression and low tumoricidal activity and promotes tissue remodeling and angiogenesis [41]. Buxton et al. reported the angiogenesis-promoting activity of extracellular NM23 protein in breast cancer [44]. In most human tumors, TAM infiltration is associated with poor prognosis, as seen in Hodgkin's disease [45]. Collectively, these results indicate that NM23 protein in extracellular environment activates monocytes and induces the tumor-promoting inflammatory cytokines and the immunosuppressive cytokines in normal PBMNC. These results also show that extracellular NM23-H1 could offer tumor cells an environmental condition convenient for their growth/survival through the cytokine production of normal PBMNC, which in turn might contribute to the poor outcome of patients with elevated serum levels of NM23-H1 protein (Figure 3).

Although rNM23-H1 induced various cytokines in both normal and leukemic PBMNCs, it promoted only the growth/survival of AML cells but not normal PBMNCs and rather stimulated its apoptosis. Moreover, rNM23-H1 protein did not have any effects on the growth/survival of normal endothelial cells (HUVECs) and various tumor cell lines (leukemia, lymphoma, neuroblastoma, and lung) [34]. NM23 proteins promoted the induction of various cytokines in the normal monocytes but not in monocytic leukemia cell lines (THP-1 and U937). Therefore, the survival-inhibiting activity of rNM23 might be specific for normal monocytes. These results indicated that rNM23 induced TAM-like functions but did not increase the growth and survival of normal monocytes, in contrast to AML cells. Taken together, these observations suggest that extracellular NM23-H1 may play an important role in the malignant progression of leukemia through normal monocytes (Figure 3).

### 3.3. Effects of Extracellular NM23 Protein on the *In Vitro* Growth/Survival and Differentiation of Other Hematopoietic Cells

Willems et al. [22] previously reported that NDPK/NM23 was present in normal human plasma and that NDPK activity correlated with hemoglobin levels, indicating the presence of NM23 in plasma as a consequence of red blood cell lysis. Moreover, they reported a modulating effect of extracellular NM23 proteins on the terminal stages (CD34^+^CD38^+^ progenitor cells) of normal hematopoietic differentiation [46]. More erythroid burst-forming units (BFU-E) and fewer macrophage colonies (CFU-M) were observed in cultures containing any of the NM23 isoforms examined, even the enzymatically inactive H118N mutant of NM23-H1. They suggest that fairly high concentrations of NM23 constitutively present in plasma/serum could have a physiologic role in supporting erythropoiesis and inhibiting excessive macrophage formation. It is interesting that extracellular NM23 serves as an alarm molecule for informing on red blood cell lysis and as a supporting molecule for normal erythropoiesis. We also reported that extracellular NM23 could inhibit the erythroid differentiation of human leukemia cell lines (K562, HEL, and KU812) without any effect on proliferation [47] and that serum NM23-H1 levels in AML-M6 (acute erythroleukemia classified by FAB (French-American-British) classification) were especially high and significantly higher than that in other FAB subtypes of AML [32]. The elevated levels of extracellular NM23 in AML-M6 might function as proliferation-supporting molecules of erythroleukemia cells as in normal erythropoiesis. In contrast to erythropoiesis, extracellular NM23-H1 seems to be inhibitory to the growth/survival of normal monocyte lineage cells [46]; however, it could promote the growth/survival of primary cultured AML-M5 (acute monocytic leukemia classified by FAB classification) cells [33]. Serum NM23-H1 levels in AML-M5 were higher than that in the other FAB subtypes of AML except AML-M6 [32]. Taken together, these results suggest that an elevated serum level of NM23-H1 protein in AML affects the biological properties of normal hematopoietic cells and leukemia cells of specific lineages and specific differentiation stages and causes poor prognosis.

## 4. Extracellular NM23-H1 Protein as a Potential Prognostic and Therapeutic Target for AML

Recent advances in genome technologies and the ensuing outpouring genomic information-related cancer have accelerated the conversion from a genome discovery into a tangible clinical endpoint. Successful examples of translating cancer genomics into therapeutics and diagnostics show the importance of establishing the biological relevance of a cancer genomic discovery in realizing its clinical potential [48]. NM23-H1 plays complex roles in the development of diverse cancers including carcinoma, high-grade lymphomas, and AML. As has been mentioned, in the case of AML and lymphomas, serum NM23-H1 protein is elevated with highest levels correlating with poorest prognosis. Moreover, the data of Lilly and colleagues [40] and our recent studies [33, 34] strongly indicate that extracellular NM23-H1 can act as a tumor-derived growth/survival factor in AML (Figure 3). These findings suggest an important biological role of extracellular NM23-H1 in the malignant progression/poor prognosis of leukemia and a potential therapeutic target for these malignancies. 

 As shown in Figure 3, extracellular NM23-H1 derived from tumor (AML) cells generates a supportive microenvironment convenient for their growth/survival of primary AML cell through cytokine production of AML cells and normal PBMNC; therefore, the reduction of extracellular NM23-H1 protein concentration or inhibitors of its action should be evaluated for therapeutic potential to combat these malignancies. Although it might be a useful technique to reduce the serum level of this protein using therapeutic filtration devices such as Hemopurifier that selectively target the removal of immunosuppressive proteins from the entire circulatory system [49], it will be very important to reveal the signal transduction pathways induced by extracellular NM23-H1 protein to increase growth/survival of AML cells. 

### 4.1. NM23 Receptors

Lilly et al. [40] recently reported the heterogeneity in the ability of AML samples to bind and respond to extracellular NM23-H1 and offered evidence that binding was essential for improveing survival. These data imply that some AMLs express an NM23-H1 receptor whereas others do not. It is interesting to examine the receptor molecules for extracellular NM23-H1 on AML cells. Recent evidence suggests that NM23-H1 can bind to a cleaved form of Mucin1 called MUC1*, which is present on the surface of many cancer cell lines and on pluripotent stem cells [50, 51]. The binding of NM23-H1 to MUC1* was reported to result in dimerization of MUC1*, and subsequent activation of the MAPK pathway to increase proliferation of the breast cancer cell line T47D [50]. Although these studies indicate that MUC1* can act as a receptor for NM23-H1, Lilly et al. [40] showed that cell surface binding to AML cells is independent of MUC1*, and therefore an alternative receptor must be present on these cells. Further studies should investigate the relationship between NM23-H1 binding and responses to AML therapies and aim to determine the nature of the NM23-H1 receptor in AML, which may provide a novel target for adjunctive therapies.

 Apetoh et al. [52] identified Toll-like receptor 4 (TLR4) ligand, high-mobility group box 1 (HMGB1) alarmin protein from dying tumor cells. This indicated that the molecule from tumor cells elicits an immune response involving the induction of inflammatory cytokines in a TLR4-dependent fashion. Reportedly, a number of endogenous proteins bind and stimulate TLR4: heat-shock protein (HSP) 60, HSP70, oxidized LDL, surfactant protein A, hyaluronan breakdown product, fibronectin, and *β*-defensin-2 [53]. The mechanism by which extracellular NM23-H1 protein induces various inflammatory cytokines in normal monocytes and AML cells (Figure 3) is unknown. It will be interesting to determine whether extracellular NM23 binds and stimulates TLR4-like HMGB1.

### 4.2. Small Molecules Regulating NM23 Functions

The downstream signaling pathway induced by the extracellular NM23-H1 also would be a potential therapeutic target for AML. We previously reported that inhibitors of MAPK/STAT3 activity suppressed the NM23-induced growth/survival of AML cells [33]. Various inhibitors are now under development, since MAPK and STAT3 signaling activations and tumor-induced inflammatory conditions are widely observed in malignant progression [54–57]. These agents might have potential to improve treatment for AML patients with a poor treatment outcome predicted by measuring serum levels of NM23-H1. 

 NM23 has a large number of functions, including NDPK activity [1, 58, 59], a transcription factor PuF for the c-Myc promoter [60], and protein kinase activity [61, 62]. Coincident with these biological characterizations, the NM23 proteins are postulated to participate in multiple biochemical activities and associations. However, it is unknown how one small molecule like NM23 exerts these many functions. Nm23-H1 and H2 form homo- and heterohexamers. It may allow the formation of a variety of isoenzymes with subtly different functions. Moreover, NM23 proteins have been reported to be associated with other proteins; transcription factors such as the retinoic acid receptor-related orphan receptor a and the retinoic Z receptor b [63], the heat shock protein Hsc70 [64], telomeres [65], Epstein-Barr virus (EBV) nuclear protein EBNA-3C [66], prune [67], and a low-molecular-weight GTPase Rad [68, 69]. These protein:protein interactions involving NM23 protein might light on studies of the multifunction observed in NM23 protein. Although we have not yet had any evidence showing NM23-interacting proteins in extracellular conditions, development of small molecules, which can modify the protein:protein interactions, may provide a novel therapeutic tool to target malignant AML. 

 EBV is an oncogenic virus associated with a number of human malignancies including Burkitt lymphoma and lymphoproliferative disease. A subset of latent EBV antigens is required for mediating immortalization of primary B-lymphocytes. EBNA-3C is one of the six latent proteins essential for EBV transformation of B-lymphocytes and interacts specifically with the NM23-H1 protein. Moreover, EBNA-3C reverses the ability of NM23-H1 to inhibit migration of Burkitt lymphoma and breast carcinoma cells. Therefore, the NM23-H1 is identified as a cellular target for EBNA-3C. NM23-H1 is predominantly localized in the cytoplasm in B-lymphocytes, while NM23-H1 is predominantly nuclear and colocalized with EBNA-3C in EBV-transformed B-lymphoblastoid cell lines and in B cells transfected with EBNA-3C [66]. These results indicate that EBV may influence the subcellular localization and function of NM23-H1 in infected cells. These results also suggest that it would be important to examine the subcellular localization of NM23-H1 protein overexpressed in leukemia and lymphoma. Zhu et al. have reported an interesting small molecule, named stauprimide, that increases the efficiency of the directed differentiation of mouse and human embryonic stem cells (ESCs) in synergy with defined extracellular signaling cues, such as activin A [70]. Using an affinity-based approach, NM23-H2 was identified as the biological target of stauprimide. By binding to NM23-H2, stauprimide inhibits NM23-H2 nuclear localization, which, in turn, represses c-Myc expression, because NM23-H2 in nucleus functions as a transcription factor for c-Myc [71]. This study points to an important role for stauprimide in modulating the subcellular localization and function of NM23-H2. The amount of intracellular NM23-H1 and NM23-H2 is inversely correlated with differentiation, and NM23 overexpression is considered to function as a differentiation suppressor in AML cells. Therefore, the suppression of the NM23's function by stauprimide should be evaluated for differentiation inducing therapy for AML.

 Expression of the human isoforms, NM23-H1 and NM23-H2, is thought to be inversely associated with metastatic potential of a variety of cancers [72–74]. While NM23-H1 is strongly associated with metastasis mechanisms in many tumors, NM23-H2 is not [75–77]. The products of these two genes, NDPK-A and NDPK-B, were named for their function as nucleoside diphosphate (NDP) kinases. NDPK-B is elaborated into the extracellular environment by the breast carcinoma cell line MDA-MB-435s as well as other cells derived from solid tumors such as colon, lung, and prostate [27]. The presence of NDP kinase activity on the surface and external environment of cancer cells that exist in the milieu of apoptosis and necrosis provides effective mechanism for regenerating extracellular purines. Buxton et al. have shown an attractive evidence to support secreted extracellular NM23-H2's (sNDPK-B) putative role in promoting metastasis [44]. The authors have provided evidence for a nucleotide-dependent regulation of angiogenesis by breast cancer of secreted extracellular NM23-H2 [27, 44, 78–80]. This can be mechanistically related to extracellular nucleotide elevation and subsequent activation of nucleotide receptors to regulate cancer growth and tumor angiogenesis [44]. These findings show that pathologically secreted NM23-H2 and its regulation of extracellular nucleotides utilize P2Y receptors to stimulate angiogenesis [44, 78]. These results also represent new therapeutic targets for antiangiogenic therapies to benefit patients. Furthermore, the authors have shown a number of inhibitors for extracellular NDPK-B activity [80]. Catechin gallates (EGCG, ECG), theaflavins, and ellagic acid (EA) are shown to inhibit NDPK-B completely with the rank order of potency: EA>theaflavins>EGCG>ECG. These compound, are known to suppress cancer cell proliferation, inhibit invasion into Matrigel, and inhibit angiogenesis [78, 80]. EA is a potent NDPK-B inhibitor that may potentiate the suppression of metastasis and thus may be useful agents to use in conjunction with traditional chemotherapy or angiogenesis inhibitors such as bevacizumab (Avastin). Using EA, we tried to inhibit the function of NM23/NDPK as a differentiation suppressor in AML cells for increasing the efficiency of the directed differentiation of AML cells in synergy with *all-trans*-retinoic acid (ATRA). EA enhanced the ATRA-induced differentiation and the apoptosis of human acute promyelocytic leukemia cell lines [81]. These results might have implications for the incorporation of anti-NM23 agents such as EA into therapeutic intervention against leukemia and possibly other hematologic malignancies overexpressing NM23.

 In conclusion, NM23 has a large number of biological functions including growth/survival-promoting activity for AML cells. Inhibitors of NM23 expression and its actions might hold promise for the treatment of AML. Especially extracellular NM23-H1 represents an important role in the malignant progression of leukemia. Therefore, its functional inhibitors and its downstream inhibitors for signaling pathways activated by extracellular NM23-H1 should be evaluated for their potential treatment as an approach to these malignancies.
